# Cadmium Removal from Giant Squid (*Dosidicus gigas*) Hydrolysate in Fixed-Bed Columns Packed with Iminodiacetic Resin: Tools for Scaling up the Process

**DOI:** 10.3390/ijerph19010442

**Published:** 2021-12-31

**Authors:** Carolina Calderón, Marcela Levío-Raimán, M. Cristina Diez

**Affiliations:** 1 Doctoral Program in Sciences of Natural Resources, University of La Frontera, Temuco 4780000, Chile; carolina.calderon@ufrontera.cl (C.C.); marcela.levio@ufrontera.cl (M.L.-R.); 2 Biotechnological Research Center Applied to the Environment (CIBAMA-BIOREN), University of La Frontera, Temuco 4780000, Chile; 3 Chemical Engineering Department, University of La Frontera, Temuco 4780000, Chile

**Keywords:** cadmium removal, iminodiacetic resin, hydrolysate protein, fixed-bed column, Thomas model

## Abstract

Giant squid hydrolysate (GSH) elaborated from different batches from a fishing company was evaluated for cadmium removal. Fixed-bed column packed with iminodiacetic resin as adsorbent was used. GSH solution at different cadmium concentrations were fed in the fixed-bed column and breakthrough curves were evaluated. A high degree of metal removal from the solution was achieved and the saturation point (C_e_/C_0_ ≤ 0.8) was achieved more quickly at higher concentrations of cadmium. The maximum capacity of adsorption (q_0_) was obtained using the Thomas model, where 1137.4, 860.4, 557.4, and 203.1 mg g^−1^ were achieved using GSH with concentrations of 48.37, 20.97, 12.13, and 3.26 mg L^−1^, respectively. Five cycles of desorption of the resin with HCl (1 M) backflow and regeneration with NaOH (0.5 M) were also evaluated, where no significant differences (*p*-value > 0.05) were observed between each cycle, with an average of 935.9 mg g^−1^ of q_max_. The *in-series* columns evaluated reached a total efficiency of 90% on average after the third column in GSH with a cadmium concentration of 20.97 mg L^−1^. This kind of configuration should be considered the best alternative for cadmium removal from GSH. Additionally, the chemical composition of GSH, which was considered a quality parameter, was not affected by cadmium adsorption.

## 1. Introduction

The giant squid (*Dosidicus gigas*) represents the most important squid fishery species in the world, accounting for 15% of global volumes from the total capture of mollusks [1]. Mollusks, such as giant squid, can be affected by absorbing and accumulating cadmium in their tissues and digestive glandules. They can assimilate more than 50% of metals present in water in a particulate or dissolved form [2]. High cadmium content has been found in the digestive glands (levels from 57 to 509 mg kg^−1^ of dry weight) and gills (levels from 6.5 to 17 mg kg^−1^ of dry weight) [3] of these animals.

Giant squid is of increasing economic interest in many countries, such as Chile, Peru, China, Mexico, and Japan, due to its high-value proteins and lipids. However, during its processing, up to 40% of this mollusk’s body weight is wasted in the form of by-products, including heads, viscera, backbones, ink, skin, fins, arms, tentacles, and unclaimed mantle. All these by-products can be turned into protein hydrolysates due to their high levels of protein, low molecular weight, high digestibility, high water holding capacity, and high-water solubility. This can be used to enhance animal nutrition (piglets, poultry, aquaculture, pet food).

As mentioned above, added-value products elaborated from giant squid by-products are contaminated with high levels of cadmium [3,4], causing a high level of risk for their consumers.

Cadmium is found in GSH as a free ion (Cd^2+^) or as an ionic complex associated with other organic and inorganic substances, such as metallothionein protein. This is a low-molecular-weight protein that is responsible for capturing and mobilizing cadmium within organs, such as the liver and kidneys [5].

In this context, the adsorption process has been widely studied and is reported to be a highly efficient and eco-friendly method for heavy metal removal. Resins are one of the most well-known adsorbents for cadmium removal. Resins are mainly composed of synthetic polymers functionalized with organic compounds that can exchange their mobile ions for ions of similar charge from the surrounding medium [6]. Despite resins being used primarily for cadmium removal from wastewater [7,8], they have also been used to remove cadmium from food, such as squid sauce [4] and squid liver [9].

The main advantages of the adsorption process are the reusability, low operating costs, improved selectivity for the specific metals of interest, short operation time, and the lack of production of secondary compounds [10].

As an effective alternative, iminodiacetic resin for cadmium removal from giant squid hydrolysate was demonstrated by Calderón et al. [11], which was the first approach in this field that addressed and proposed solutions for food application. A batch assay comparison between a standard solution of cadmium and GSH samples revealed high variations in adsorption of cadmium, which was influenced highly by the complexity of the matrix. In this sense, is important to improve the adsorption process used to test continuous systems. Additionally, fixed-bed columns were reported as more advantageous than the batch method because of their simplicity of operation, faster adsorption time, and easy scale-up process from the lab [11,12].

The purpose of this study was to evaluate the use of fixed-bed columns for the removal of cadmium from a giant squid hydrolysate (GSH) using iminodiacetic resin as an adsorbent that does not affect the nutritional components of this product in order to improve the scalability of the process.

## 2. Materials and Methods

### 2.1. Adsorbent

**Figure 1:**
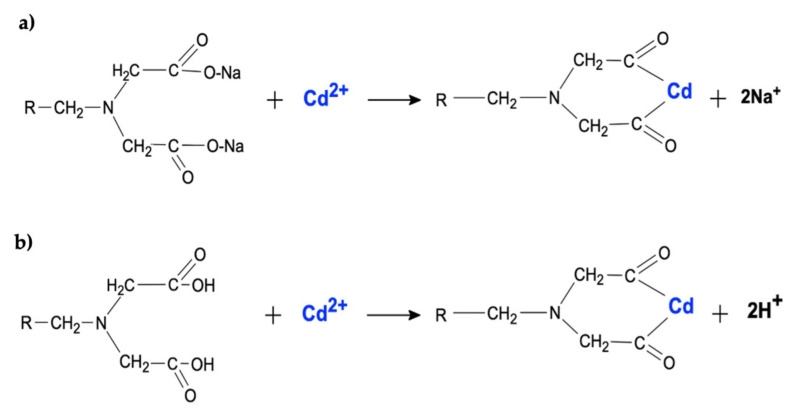
Iminodiacetic acid is the functional group of resin and the mechanism of chelating of cadmium by the (**a**) Na form and (**b**) H form.

A commercial resin provided by Dimerco^®^ was used for cadmium removal. This resin is a macroporous styrene-divinylbenzene polymeric matrix with donor atoms that can form chelate complexes with metallic ions, the functional group of which is based on iminodiacetic acid (Figure 1). The resin has a specific surface BET of 21.5 m^2^ g^−1^, a pore volume of 0.036 cc g^−1^, a pore diameter of 1.04 nm (obtained by Calderon et al. [11]), and a total exchange capacity of 30 g of cations L^−1^.

### 2.2. Giant Squid Hydrolysate

Giant squid hydrolysate (GSH) is a liquid product that contains soluble proteins, a lipidic fraction, and suspended solids corresponding to the non-hydrolyzed fraction. Giant squid hydrolysate (GSH) was obtained via an enzymatic hydrolysis process using giant squid by-products and was elaborated in a seafood plant processing located in the Bío-Bío region of Chile at a real scale (2500 kg per batch). The giant squid by-product consisted mainly of a mix of muscle tissue (mantle, tentacles, fins, and skin) and digestive glands. Considering that high cadmium content was found in digestive glands, four GSH batches were prepared with different proportions of digestive glands, as shown in Table 1. Giant squid by-products were ground and hydrolyzed using an enzymatic method (commercial protease, with a dosage of 0.01% of the total protein content) for 1 h while stirring (200 rpm) at 50 °C. Subsequently, the soluble fraction was sieved in order to remove the solids from the cartilage and bones, heated for pasteurization, and then centrifugated to remove the major fat contents. After each GSH batch, samples were taken for the cadmium removal assays. For GSH characterization, after each batch, samples of GSH were dried in a spray dryer (Niro Mod. Mobile Minor^TM^).

### 2.3. Column Design at Lab Scale

In order to make the cadmium adsorption representative of the industrial process of GSH production, a reference flowrate of 2.8 m^3^ h^−1^ was considered for the column design on the lab scale. The following variables were considered for the column design: linear velocity, space velocity, and expansion of the resin pack.

Linear velocity, defined by the path of the fluid through the transversal section of the column per unit of time [13], is represented in Equation (1):

(1) $$\mathrm{LV}=\frac{Q}{A}\;$$

where *Q* is the flow rate (cm^3^ s^−1^); *A* is an area of a cross-section (cm^2^), and LV is the linear velocity (cm s^−1^).

Space velocity is frequently used in ion exchange and adsorption operations in fixed beds and is frequently called “relative volumetric flow rate” (Q*_rel_*). The most common unit of Q*_rel_* is bed volumes per hour (BV/h) [13], described in Equation (2):

(2) $$\mathrm{EV}=\frac{Q}{V_{r}}$$

where *Q* is the flow rate (cm^3^ s^−1^) and *V_r_* corresponds to the volume of the adsorbent (cm^3^).

In order to facilitate the backwash operation described in Section 2.4, it was necessary to calculate the expansion percentage of the adsorbent into the column. The supplier of the resin recommended an expansion of 50–75%. The expansion is represented by Equation (3):

(3) $$E\;\left(\%\right)=\left(\frac{H-h}{h}\right)\times 100$$

where *H* is the height of the column (cm), *h* is the height of the resin (cm), and E corresponds to the expansion (%).

**Figure 2:**
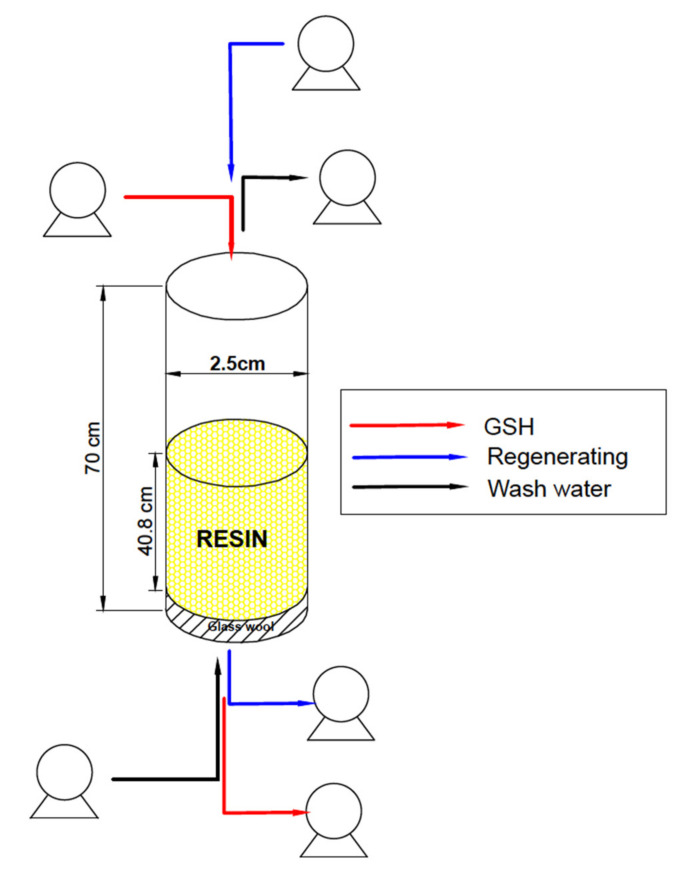
Column designed with a length of 70 cm and an internal diameter of 2.5 cm packed with 140 g of iminodiacetic resin equivalent to a volume of 200 cc. A 71.5% of expansion rate was considered.

All these variables were put into an objective function using SOLVER^®^ in Microsoft Excel in order to check their compliance with all the restrictions and optimize the column operation service. The resulting column had a length of 70 cm, an internal diameter of 2.5 cm, 140 g of iminodiacetic resin, a linear velocity of 0.475 cm s^−1^, a space velocity of 0.012 s^−1^, and a 71.5% expansion rate (Figure 2).

#### 2.3.1. Operation of the Fixed-Bed Column

Stainless steel columns, the dimensions of which were obtained from Section 2.3 (70 cm length and 2.5 cm internal diameter), were packed with 140 g of iminodiacetic resin (200 cc) and operated in a continuous system. Before eluting the GSH, the columns packed with iminodiacetic resin were preconditioned with water at 55 °C for 10 min. Liquid GSH at 55 ± 2 °C and pH 6.2 with different cadmium concentrations according to each batch described in Table 1 was pumped from the top of the column at a flow rate of 0.14 L min^−1^ using a peristaltic pump. At the outlet of the column, the treated GSH sample was collected at regular time intervals. The experimental conditions that influenced the adsorption of cadmium in the fixed-bed column were evaluated using breakthrough curves. The saturation point was determined using *C_e_*/*C*_0_ ≥ 0.8, where *C_e_* and *C*_0_ (mg L^−1^) are the cadmium concentrations in the effluent and influent, respectively. The evaluation of the column performance was conducted by plotting the relation between the cadmium concentration in the effluent with the cadmium concentration in the influent (*C_e_*/*C*_0_) as a function of time (minutes). The cadmium concentration was measured using an atomic absorption spectrophotometer (AAS). All experiments were carried out in triplicate. Additionally, the total adsorption capacity of the column (q_total_) at a given pH, inlet concentration, and flow rate was evaluated experimentally and was calculated using Equation (4):

(4) $$q_{\mathrm{total}}=\int_{t=0}^{t=total}\left(C_{0}-C_{t}\right)\;dt$$

#### 2.3.2. Modeling of Cadmium Adsorption in the Fixed-Bed Column

The experimentally obtained breakthrough curves were fitted using the Thomas model [14] to calculate the adsorption rate and solid-phase concentration of cadmium from continuous mode studies. The nonlinear form of the Thomas model can be expressed as described below in Equation (5):

(5) $$\frac{C_{e}}{C_{0}}=\frac{1}{1+\exp\left[\frac{K_{Th}\cdot \left(q_{0}\cdot m-C_{0}\cdot V\right)}{F}\right]}$$

The linearized form of the Thomas model can be expressed as follows in Equation (6):

(6) $$\ln\left(\frac{C_{0}}{C_{e}}-1\right)=\frac{K_{Th}\cdot q_{0}\cdot m}{F}-K_{Th}\;\cdot \;C_{0}\cdot t$$

where *C_e_* is the outlet cadmium concentration (mg L^−1^), *C*_0_ is the initial cadmium concentration in the influent (mg L^−1^), *K_Th_* is the Thomas model rate constant (mL min mg^−1^), *q_0_* is the equilibrium cadmium from GSH adsorbed per gram of resin (mg g^−1^), *m* is the mass of adsorbent (g), *V* is the effluent volume (mL), and F is the flow rate (mL min^−1^). The constants K_t_ and *q*_0_ were determined from the linear form of the Thomas model (Equation (6)).

### 2.4. Column Desorption, Regeneration, and Reusability Assay

For the cadmium desorption study, the column for cadmium adsorption of GSH from batch 2 (20.97 mg L^−1^) was used due to this cadmium concentration being more frequently found in by-products of giant squid. As an elution reagent, HCl (1 M) was used and fed into the backflow at a flow rate of 0.1 L min^−1^. The eluted solution was collected at regular time intervals for 30 min and the cadmium content was measured using AAS. After the desorption step, NaOH was used for resin regeneration via conversion to the Na^+^ form by eluting NaOH 0.5 M at a flow rate of 0.5 L min^−1^ for 20 min. Subsequently, the column was washed thoroughly using deionized water and reused for each cycle of cadmium adsorption.

The sequence of desorption–regeneration followed by a wash was considered one cycle. The evaluation of the adsorbent reusability was conducted after five repeated cycles. The saturation points and desorption curves of each cycle were evaluated using the relation *Ce*/C_0_ as a function of time (min). Additionally, each cycle was evaluated using the Thomas model and the total capacity of cadmium adsorption was determined as described in Section 2.3.1 and Section 2.3.2.

### 2.5. In-Series Columns System Evaluation

Stainless steel columns prepared and operated as described in Section 2.3.1 were disposed *in series*, and liquid GSH from batch 2 (20.97 mg L^−1^) was added at the top of the first column, which then feed the subsequent columns. Each column was operated for 60 min. The cadmium concentration at the end of the first column was considered the initial concentration of the next column. Cadmium removal (%) was calculated using Equation (7), where *C*_0_ is the initial concentration and *C_T_* is the cadmium concentration after the third column operation:

(7) $$\mathrm{Cadmium}\;\mathrm{removal}\;\left(\%\right)=\left(\frac{C_{0}-C_{T}}{C_{0}}\right)\cdot 100$$

The characterization of GSH samples before and after cadmium removal in fixed-bed columns disposed of *in series* was made. Before the analyses, samples were spray dried and the results are expressed as the dry weight. The chemical composition, mineral profile, and aminoacidic composition of GSH before and after the *in-series* adsorption process using iminodiacetic resin were determined (Section 2.7).

### 2.6. Statistical Analysis

Statistical significance was assessed using one-way ANOVA with Tukey’s test using the software package SPSS/PCTM version 22.0 (SPSS Inc., Chicago, IL, USA). The *p*-values less than 0.05 were considered statistically significant.

### 2.7. GSH Characterization

GSH was analyzed using the following methodologies: moisture (ISO 6496/1999), protein (AOAC Method 984.13, 1994), fat (AOAC Method 920.39, 1920), total ashes (ISO 5984:2002), and pepsin digestibility (AOAC Method 971.09). The mineral profile was determined using an atomic absorption spectrophotometer (AAS) (Perkin-Elmer model PinAAcle 900-H) and plasma emission spectroscopy (inductively coupled plasma (ICP)). The aminoacidic profile was analyzed using HPLC (626 LC System, Column (AccQ—Tag)) and a UV-visible detector. Moreover, before and after saturation of the columns with a GSH solution, the iminodiacetic resin packed in a bed column was dried at 60 °C for 24 h and used for resin observation using scanning electronic microscopy (SEM) (Model SU-3500 Hitachi, Tokyo, Japan).

## 3. Results and Discussion

### 3.1. Breakthrough Curves

**Figure 3:**
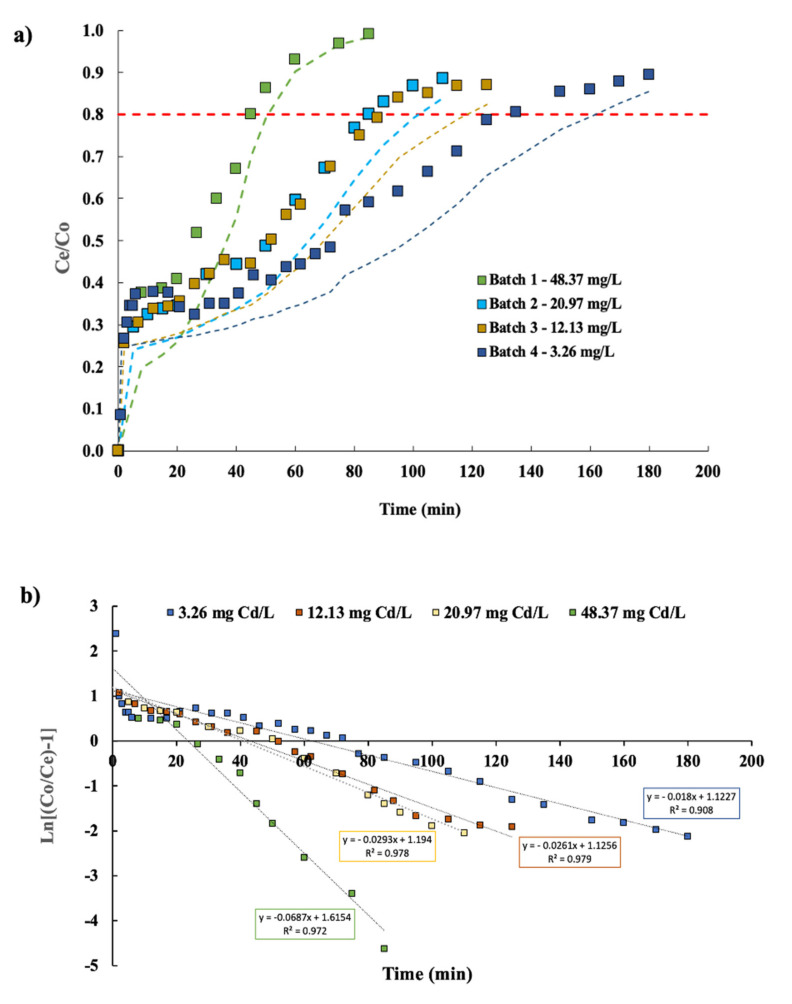
(**a**) Effect of the influent concentration on the breakthrough curves for cadmium adsorption by a fixed-bed column packed with resin as an adsorbent until saturation (C/C_0_ < 0.8). The symbols represent the experimental data and the dotted line represents the data fitted to the Thomas model. (**b**) Linearized representation of the Thomas model at the different initial concentrations of cadmium in the GSH solutions represented by the GSH solution with 3.26 mg L^−1^, 12.13 mg L^−1^, 20.97 mg L^−1^, and 48.37 mg L^−1^ cadmium concentrations. Both representations were performed with a flow rate of 0.14 L min^−1^, pH of 6.2, adsorbent mass of 140 g, and operating temperature of 55 °C.

The cadmium concentrations in the GSH varied according to the proportion of raw material (giant squid by-product), where the amount (%) of digestive glands had a relevant influence on the content of cadmium during the process (Table 1), with a correlation coefficient (R^2^) of 0.975. Figure 3a displays the breakthrough curves constructed at different cadmium concentrations present in the GSH. The adsorption breakthrough curves showed two clearly differentiated phases. As the concentration of cadmium in the GSH increased, the column saturated more quickly. The earliest breakthrough point at a higher cadmium content in the GSH (48.37 mg L^−1^) was observed at 45 min. The binding sites became more quickly saturated in the column and this indicated that an increase in the cadmium GSH inlet concentration could modify the adsorption rate through the bed [14]. On the other hand, when the cadmium content in the GSH was the lowest (3.26 mg L^−1^), the column saturation was three times slower (135 min).

In this context, Nazari et al. [15] observed that a high concentration gradient can increase the intensity of transport inside the pores (i.e., diffusion coefficient), thereby accelerating the adsorption process and saturation. A gradual saturation of binding sites was also observed by Sasaki et al. [4], where the cadmium concentration of a fish sauce increased linearly with elution volume passed through a resin with ethylenediamine as the functional group.

### 3.2. Thomas Modeling in Columns Assays

The experimental adsorption breakthrough curves for cadmium were constructed and compared using the breakthrough curves predicted according to the Thomas model, as represented by Figure 3a by dotted lines. Figure 3b presents the linearization of the Thomas model and the experimental data, where high correlation coefficients (R^2^) were obtained with values of 0.972, 0.977, 0.979, and 0.908 for 48.37, 20.97, 12.13, and 3.26 mg L^−1^ cadmium concentrations in the GSH, respectively (Table 2). Table 2 provides the Thomas model parameters and a comparison of the quantity adsorbed (experimental and theoretical) at different initial concentrations of cadmium in GSH solutions. In general, the maximum experimental adsorption of cadmium (q_total_) was similar to the predicted maximum adsorption of cadmium (q_max_) by the model, where the highest value of cadmium adsorbed onto the resin was for an initial concentration of 48.37 mg L^−1^ of cadmium in the GSH solution, with a q_total_ of 1015.3 mg g^−1^ and q_max_ of 1137.4 mg g^−1^, thus demonstrating a major affinity of the resin for cadmium at elevated cadmium concentrations in the GSH.

With respect to the adsorption capacity, Sasaki et al. [4] obtained an estimated value of maximum capacity of 0.3 mg g^−1^ for a fish sauce with a cadmium concentration of 300 mg L^−1^ and a flow rate of 5 mL h^−1^ with chelate resin as the adsorbent according to their experimental results. Xiong and Yao [6] achieved a maximum capacity of 349 mg g^−1^; however, different conditions of cadmium concentration (200 mg L^−1^), influent flow rate, and type of eluted sample were used.

Regarding the Thomas rate constant (K_T_) shown in Table 2, an inverse relation between the cadmium concentration in the GSH and the rate constant (K_T_) was observed. Values of K_T_ were 0.0014 and 0.0055 L min mg^−1^ for 48.37 and 3.26 mg L^−1^, respectively. The same value of K_T_ of the GSH with a cadmium concentration of 48.37 mg L^−1^ and 20.97 mg L^−1^ was obtained. Thus, as the cadmium concentration increased, the K_T_ value decreased, which means that the resistance to the mass transference of the pollutant into the resin was decreased. This enhanced the adsorption capacity of the resin and, therefore, favored the intra-particular diffusion from the solution up to the solute. At the same time, the opposite effect was observed at lower cadmium concentrations in the GSH solution, which reduces the adsorption capacity (*q*_0_) of the resin and increased its resistance to the mass transference of cations.

### 3.3. Desorption and Reusability System

The regeneration of the adsorbents is one of the key factors used to assess their potential utilization in industrial scale applications, which would save operation costs for a removal system.

**Figure 4:**
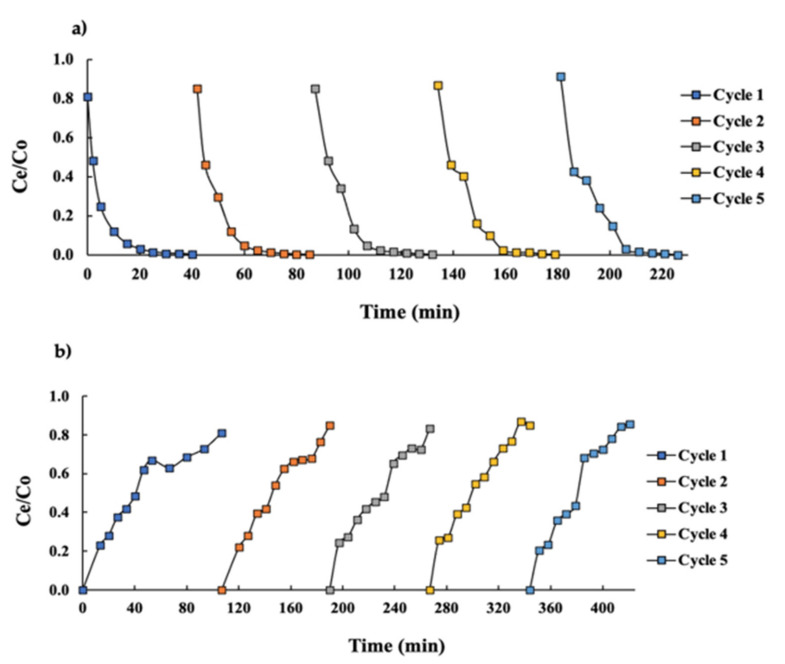
Sequences of cadmium desorption–regeneration–conversion–washing of the saturated column with the GSH solution at a 20.97 mg L^−1^ cadmium concentration, pH of 6.2, and temperature of 55 ± 2 °C. A backwash solution as a desorption agent was added at a flow rate of 0.1 L min^−1^. (**a**) Desorption of the cadmium process represented by 5 cycles. (**b**) Cycles of saturation of resin by cadmium from GSH.

The desorption system was evaluated in order to test the reusability of the resin. In this context, HCl (1 M) allowed for the removal of the cadmium chelated to the surface of the resin and was effective as a desorption agent after saturation (Figure 4a).

Cadmium desorption from a saturated column with the GSH solution at 20.97 mg L^−1^ was achieved after 18 min, removing 97% of the cadmium adsorbed in the resin. These results were corroborated by Saleh et al. [16] with a desorption efficiency between 92–100% using HCl at 0.3 M for cadmium adsorbed by a chelate resin. Additionally, and according to previous assays, better efficiency of desorption and subsequent saturation was achieved when the resin was converted to the Na form. Similarly, Taha et al. [17] achieved the best desorption efficiency with HCl and H_2_SO_4_ (1 M) as desorption agents, showing 98.9% and 100% cadmium removal, respectively, from saturated sulfonic and amino-phosphonic resins.

On the other hand, similar breakthrough points (*C_e_*/*C*_0_ = 0.8) were observed in each cycle after the desorption and regeneration process (Figure 4b). Saturation was achieved at similar times (100 min) and no significant differences (*p*-value > 0.05) were observed between each cycle of saturation–desorption–regeneration. The resin was able to remove cadmium from the GSH solution, desorbing the cadmium and regenerating correctly in five consecutive cycles.

The adsorption capacity (*q*_0_) was evaluated in each cycle, where no significant differences were observed (*p* > 0.05). Moreover, an average q_max_ value of 945 mg g^−1^ between the five cycles was obtained. In terms of the loss in the adsorption capacity of the resin for cadmium removal, no losses were detected after each cycle. This might be due to the unremarkable mass of adsorbent lost during the adsorption–desorption process. These results indicated that the iminodiacetic resin packed in a fixed-bed column offers the potential to be used repeatedly in cadmium adsorption studies without any detectable loss in the total adsorption capacity over five consecutive cycles. It should be noted that there have been no reusability studies performed with iminodiacetic resin removing cadmium from this type of matrix (GSH).

### 3.4. In-Series Columns System Evaluation

**Figure 5:**
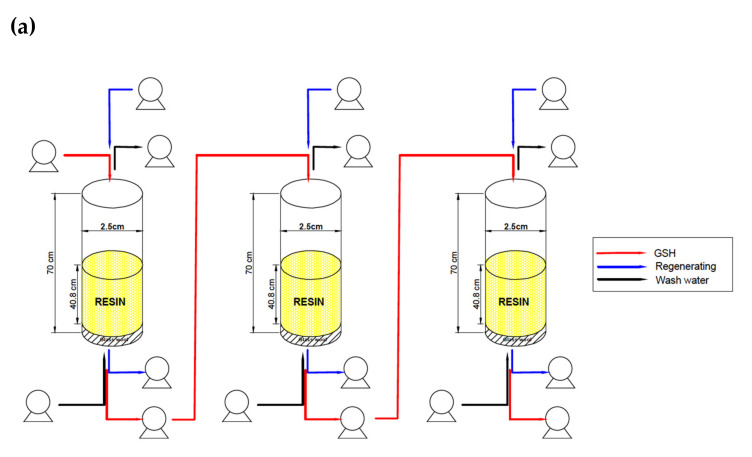

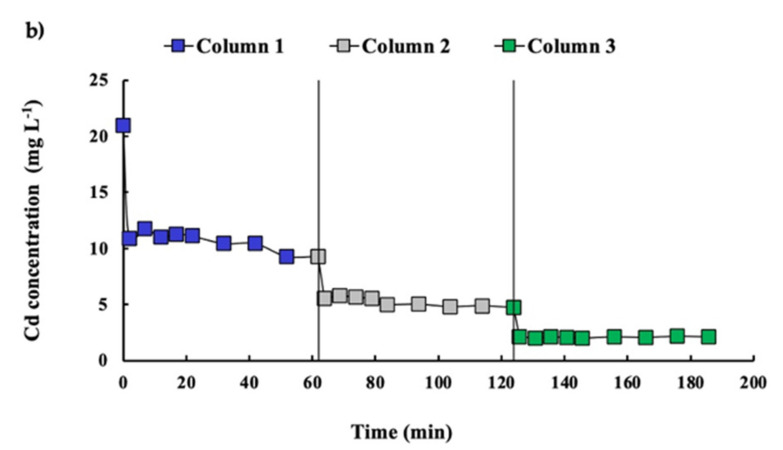
(**a**) Schematic representation of the three columns connected *in series*. (**b**) cadmium concentration performed in the column assay connected *in series*. The initial cadmium concentration in column 1 was 20.97 mg L^−1^. The final concentration of one column was the inlet concentration of the next column. The final concentration was 2.10 mg L^−1^.

Three columns connected *in series* were packed with resin and the cadmium removal from the GSH solution at 20.97 mg L^−1^ of cadmium was evaluated and is shown in Figure 5. After the first column operated for 60 min, the cadmium concentration decreased from 20.97 to 9.0 mg L^−1^, followed by the second column with a decrease from 9.0 to 4.8 mg L^−1^ and a decrease of cadmium concentration of GSH after the third column from 4.8 to 2.1 mg L^−1^ (Figure 5)). Regarding the total removal, the three columns reduced the cadmium concentration of the GSH from 20.97 mg L^−1^ to 2.10 mg L^−1^. In this sense, the three steps resulted in a better cadmium removal system than one, with a total removal of 90%. Accordingly, triple the resin in one column could be used as another alternative. In this regard, resins allowed for the removal of higher volumes of the pollutant and may be reused several times. Otherwise, the first column favors the cadmium removal, where the cadmium concentration fed in the column was higher. This can be explained by changes in the surface adsorption properties during the binding of ions on the inner and outer surfaces, which is more intensive at higher initial concentrations [18]. Speciation of the metal at pH 6.2 also favors cadmium entrapment by the 2Na^+^ present in the resin structure (iminodiacetic acid).

On the other hand, results of 98% and 99% cadmium removal were obtained by Elbadawy et al. [19] from a standard solution, where the column method was considered more efficient and economical than the batch process for practical applications. However, high cadmium removal was achieved using an ideal matrix (standard cadmium solution) and lower flow rate conditions with a standard cadmium solution of 18.24 mg L^−1^ and 1.5 mL min^−1^, respectively. 

### 3.5. GSH Characterization

The chemical characterization of the liquid GSH samples from batch 2, which was obtained after the centrifugation step, before and after the adsorption in fixed-bed columns is shown in Table 3. The total protein content, soluble protein, and digestibility did not change significantly (*p* > 0.05) due to the total adsorption process. The main difference between the before and after the total adsorption process was related to the fat (23%) and salt (5.5%) contents (*p* < 0.05). The fat retained on the resin surface acted as a waterproofing agent, where it was able to block the surface and the active sites of the resin. In fact, the polymeric matrix of the resin (styrene-divinylbenzene) is able to capture lipophilic compounds and this resin is generally used as a package of chromatographic columns for the retention of antibiotics, toxins, etc.

**Figure 6:**
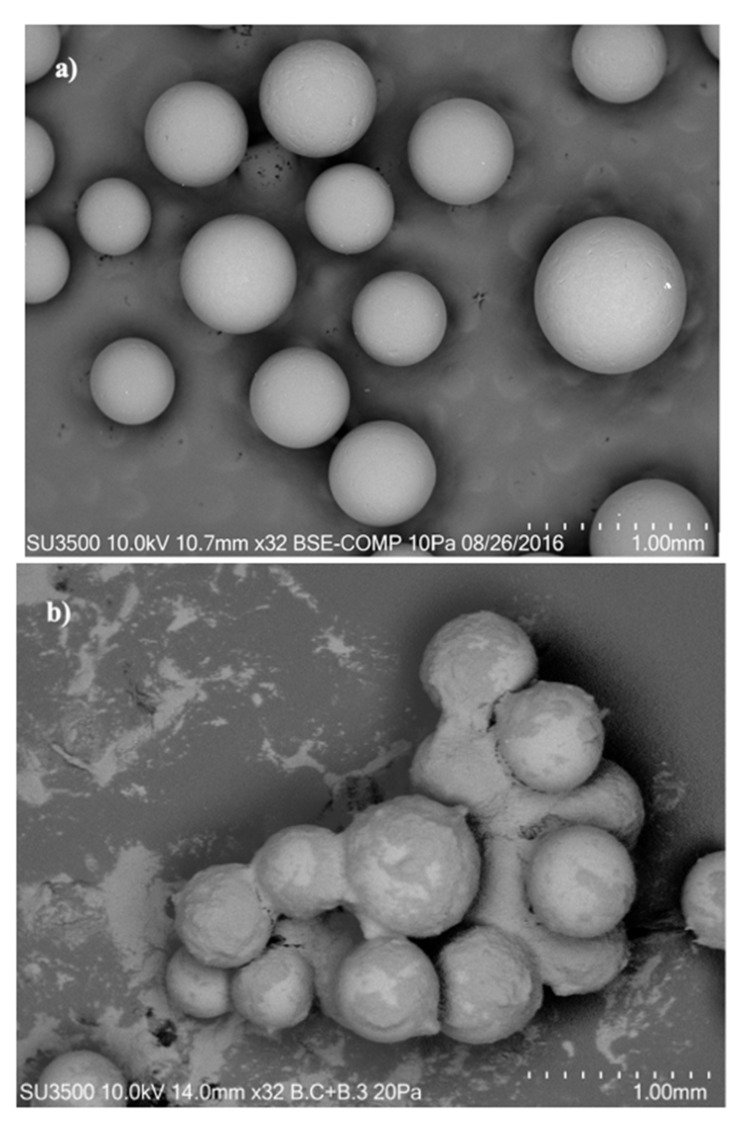
Scanning electronic microscopy (SEM) images of the resin before and after the cadmium adsorption from the GSH solution. (**a**) Resin in the original state and (**b**) resin from a saturated column. The cadmium concentration of the liquid GSH was 20.97 mg L^−1^.

On the other hand, solids also blocked the binding sites and could form a kind of fouling on the saturated resin. Figure 6 shows the SEM images captured before and after the adsorption process. Before the adsorption process, a spherically shaped resin with an average diameter of 560 µm, a uniform morphology, and a smooth surface was observed (Figure 6a). After the total adsorption process, the resin was coated with a film consisting of white spots, which was attributable to the presence of inorganic material, and a layer of lipid material on the surface of the resin (Figure 6b). Calderon et al. [11] showed that in a batch study, 44.9% of the fat content from GSH remained in resin after an adsorption treatment, meaning losses on the adsorption capacity of the resin and a faster resin saturation. In contrast, the use of adsorption fixed-bed columns connected *in series* allowed us to treat the GSH more efficiently despite the lipid layer coating the resin. In this regard, the presence of undesirable compounds in the adsorption process for cadmium removal, such as other metals, salts, and fat content, interfered with and blocked the binding sites of the resin.

Table 4 shows the mineral profile of the liquid GSH before and after the adsorption process in the fixed-bed columns connected *in series*. We can observe that the GSH had high sodium (Na) content (>16,000 mg kg^−1^) followed by magnesium (Mg) and calcium (Ca) (>600 mg kg^−1^). Cadmium and copper (Cu) were present in the GSH at concentrations of 138.8 mg kg^−1^ and 219 mg kg^−1^, respectively, and zinc (Zn) and iron (Fe) were present at concentrations of less than 80 mg kg^−1^. Sodium was predominantly present as NaCl in the GSH due to the marine habitat of the species. After the total adsorption process, a high degree of cadmium removal was obtained (90%), followed by Fe, Cu, and Na (>81%). To a lesser extent, Zn and Mg were removed (78.63 and 68.4%, respectively), followed by Ca removal (34.28%) (Table 4). In our study, no competition between cadmium and the other metals was observed, in contrast to data reported by other authors [7,20,21], who evidenced competition for the active sites of adsorbents. According to the technical specifications of the commercial iminodiacetic resin, it is particularly suitable for the removal of heavy metals (as weakly acidic chelated complexes), which are held according to the following order of selectivity: Cu >> Ni > Zn ≥ Co ≥ Cd > Fe(II) > Mn > Ca. In our study, the adsorption sequence was as follows: Cd >> Cu > Na > Fe > Zn > Mg > Ca. The greater cadmium adsorption compared to copper and other metals can be explained by the pH of the GSH (6.2), which favors cadmium adsorption, which, according to the speciation, copper requires acidic conditions (pH 2–5) to be removed easily. Moreover, the result suggests that cadmium could be released from Cd–metallothionein (Cd–MT) during the enzymatic hydrolysis process to obtain the GSH, where protein is fractioned to achieve peptides with lower molecular weight, which could facilitate greater adsorption onto the resin than other metals. Despite the high level of monovalent element (Na) present in the GSH, no inhibition of cadmium adsorption onto the resin by Na was observed. This is consistent with the study performed by Sasaki et al. [4], where they demonstrated that cadmium may bind iminodiacetic acids more strongly than Na, reflecting differences in the binding modes and spatial structures.

Regarding the nutritional or antinutritional properties of GSH as an ingredient intended for animal consumption, the elimination of other metals, such as copper, is beneficial. Copper is classified by the Association of Food Control Officials of the United States (AFFCO) as toxic, where levels between 10–40 ppm in a complete diet and levels between 100–1000 ppm are suggested for food ingredients. Low levels of toxic elements reinforce the idea of commercializing safe food. In contrast, elements such as zinc have an average requirement in diets of 50–100 ppm (depending on the species). In this sense, the decrease in this metal due to the effect of cadmium removal could imply that the formulator must supplement the deficiency. It should be noted that the addition of these protein hydrolysates such as GSH in diets does not exceed 5%; therefore, the contribution of contaminants and essential minerals will not be significant in the complete formulation.

Cadmium adsorption from the GSH placed onto fixed-bed columns connected *in series* using the iminodiacetic resin as an adsorbent did not greatly affect the content of the GSH amino acids (Table 5). On the other hand, the amino acid content did not interfere with the cadmium adsorption process. A similar result was reported by Sasaki et al. [4] where the total free amino acids did not influence cadmium removal in a squid sauce using iminodiacetic resin. Moreover, we did not observe important interference by amino acids as part of metallothionein (MT). MT possesses a highly conserved amino acid sequence that is mainly composed of up to 20 cysteine (Cys) residues [22] and metal-rich proteins containing sulfur-based metal clusters formed with Zn^2+^, Cd^2+^, and Cu^2+^ ions. In the case of GSH, predominant amino acids, such as histidine, arginine, and lysine (Table 5), have basic R groups. Therefore, they tend to bind protons, gaining a positive charge in the process, where the protonated forms predominate at physiological pH (about 7). Thus, the pH of GSH (6.2) also favors some competition for the cadmium binding between amino acids and resin.

### 3.6. Mass Balance and Analysis Data for Simulation

The design of cadmium removal columns for scaling up to an industrial scale involves several restrictions for the process to achieve the maximum cadmium removal. The columns connected *in series* removed 90% of cadmium from the GSH with a cadmium concentration of 20.97 mg L^−1^. This result, along with those obtained from the Thomas model for the maximum adsorption capacity (*q*_0_) and others provided by the industry, were used for a general prospecting of a technology on an industrial scale. In this sense, a simulation of a series of three columns was carried out on an industrial scale, feeding the columns connected *in series* with GSH at 2.8 m^3^ h^−1^ in order to observe the removal capacity of cadmium per unit of time. The amount of cadmium to be treated as waste from the process and the level of exhaustion of the resin are shown in Table 6.

Following the removal of 90% of cadmium from liquid GSH, a GSH with a cadmium concentration of 13.8 mg kg^−1^ (d.w) was obtained. In this regard, cadmium was not removed enough to comply with European regulations, where Directive 2002/32/EC establishes a maximum concentration of 2 mg kg^−1^ adjusted to a moisture content of 12% for feed ingredients. However, according to AFFCO’s official guidelines for contaminants in individual food ingredients, the suggested cadmium levels are between 5 and 500 ppm. As an example, if GSH is included as 5% of the diet, with 13 ppm of cadmium it would contribute 0.7 ppm of cadmium in the diet versus 7.0 ppm without any treatment for cadmium removal. In this sense, the decrease in cadmium levels will be beneficial by providing a competitive advantage relative to other products.

**Figure 7:**
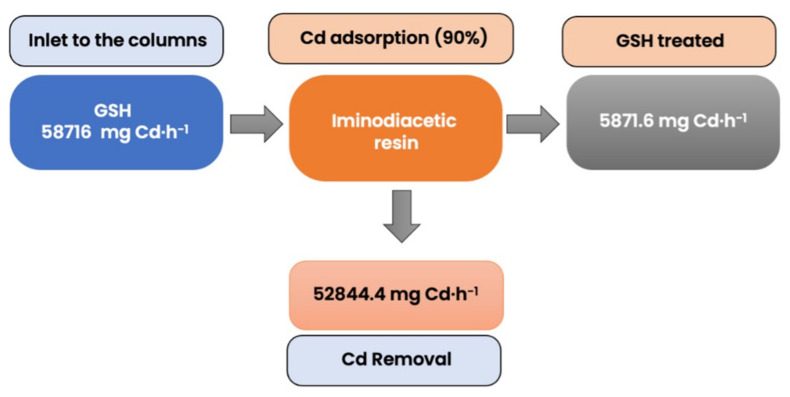
Representation of the mass balance of cadmium removal from GSH by columns *in series* with 90% cadmium removal at an industrial scale. The simulation consisted of GSH with a Cadmium concentration at the inlet of 20.97 mg L^−1^, pH of 6.20, a flow rate of GSH of 2800 L h^−1^, and a resin content of 140 kg per column.

Figure 7 shows that the effluent after the treatment had a mass of cadmium of 5871.6 mg h^−1^ and that it retained 52,844.4 mg h^−1^ of the total resin (420 kg for the three columns).

The process described in this paper generated waste effluents in the form of the HCl and NaOH washing solutions, which were contaminated with cadmium, as well as lipids, proteins, and other heavy metals. Therefore, additional treatment for the removal of cadmium, as well as the other impurities, should be considered for the wastewater of food processing plants, which will use the GSH treatment described here. In this sense, cadmium removal from wastewater has hardly been studied, and the most common technology for wastewater purification has been coagulation and flocculation, followed by sedimentation and filtration. These are also used to remove heavy metals from aqueous solutions, where coagulation destabilizes colloids by neutralizing the forces that keep cadmium apart. On the other hand, a low-cost alternative was proposed by Levío-Raiman et al. [23], where an organic biomixture packed in columns was considered a good alternative to remove metal ions.

Level of exhaustion resulted by the relation of the mass of cadmium removed by the resin used by 20 h per day and the cation exchange capacity provided by the resin supplier. In this context, the level of cadmium exhaustion in the resin reached 0.589%. On the other hand, 142 days would be necessary to saturate the column only with cadmium. This could be attributed to the presence of other metals, proteins, amino acids, and lipids due to the complexity of the GSH, which interferes with the cadmium adsorption by the resin. Physical blockage instead of chemical exhaustion via any excess of cations was observed. Another reason for the low capacity (as compared with the maximum possible cadmium sorption capacity) was the use of the columns *in series*, where the saturation was undesirable in order to keep the adsorption in equilibrium during the 60 min of operation.

## 4. Conclusions

In this study, we found that the cadmium concentration in the GSH was dependent on the amount of digestive glands as raw material and the feasibility of iminodiacetic resin for cadmium removal from GSH in continuous conditions using fixed-bed columns. Better removal was obtained with high cadmium concentrations in the GSH. Regarding the cadmium capacity of adsorption, the total capacity obtained (q_total_) from experimental data was similar to that predicted by the Thomas model (q_max_), where all the batches at different concentrations of cadmium were fitted to the model proposed (R^2^ > 0.90).

Desorption–regeneration and reusability studies demonstrated its effectiveness, where acid wash desorption (HCl) and regeneration with NaOH allowed the spent material to be reused for five consecutive cycles with no significant loss in performance.

More contact time and mass of resin into the column were needed to increase the cadmium removal. Accordingly, the study of the columns connected *in series* exhibited a total efficiency of 90% after the third column. This kind of configuration could be considered the best alternative for cadmium removal from GSH.

On the other hand, comparative chemical characterization of GSH before and after cadmium adsorption could confirm a high affinity for cadmium removal over copper and other metals. Additionally, the column saturation could be explained by a physical blockage with the removal of lipids from the GSH acting as a waterproofing agent. This was confirmed by a calculation of the level of cadmium exhaustion, where 0.589% was the obtained result.

Despite the high level of cadmium removal, it was not possible to achieve a concentration below the maximum for feed ingredients as regulated by the European Union (DIRECTIVE 2002/32/EC), which is 2 mg kg^−1^ for ingredients elaborated from squid, adjusted to a moisture level of 12%. Thus, the content of cadmium needs to be further managed in GSH elaboration by the standardization of the content of digestive glands, increasing the number of columns, or studying a semicontinuous method (batch columns).

The information generated in this study allows for the implementation and/or adoption of technology suitable for the food and feed industry.

## Figures and Tables

**Table 1:** Batches of giant squid hydrolysate (GSH) elaborated with different proportions of digestive glands at different production dates (weekly during May) and the resulting concentration of cadmium and pH of each batch.

Batch N°	Digestive Glands (%)	Cadmium Concentration (mg L^−1^)	pH
1	70	48.27	6.16
2	40	20.97	6.20
3	20	12.13	6.15
4	5	3.26	6.11

**Table 2:** The Thomas model parameters in a fixed-bed column packed with iminodiacetic resin that were found at different initial concentrations of cadmium in the GSH solution. The experimental conditions were a pH of 6.2, a flow rate of 140 mL min^−1^, and a temperature of 55 °C.

Initial Cd Concentration (mg L^−1^)	q_max_ (mg g^−1^)	K_T_ (mL min^−1^ mg^−1^)	R^2^	q_total_ (mg g^−1^)
48.37	1137.4	0.0014	0.972	1015.3
20.97	860.4	0.0014	0.978	827.1
12.13	557.4	0.0020	0.979	519.8
3.26	203.1	0.0055	0.908	200.3

**Table 3:** Characterization (%) of the GSH sample from batch 2 (20.97 mg L^−1^) before and after the cadmium adsorption in fixed-bed columns connected *in series*. The experimental conditions were a pH of 6.2, a flow rate of 140 mL min^−1^, and a temperature of 55 °C. The results are expressed as the dry weight and are the average of three repetitions.

Parameter	GSH before Adsorption	GSH after Adsorption
	(% d.w)	(% d.w)
Protein	83.9 ± 0.9 a	86.3 ± 0.9 a
Fat	7.8 ± 0.6 b	5.9 ± 0.1 b
Ash	8.0 ± 0.4 a	7.6 ± 0.1 a
Salt (NaCl)	4.8 ± 0.2 ab	3.9 ± 0.1 ab
Soluble protein	63.5 ± 2.5 a	62.6 ± 0.5 a
Digestibility (pepsin)	99.9 ± 0.0 a	99.9 ± 0.0 a

The average values and the standard error are presented (*n* = 3). Different letters refer to the significant differences (*p* < 0.05, Tukey’s test) of the mean values of each parameter before and after the cadmium removal.

**Table 4:** The mineral profile (mg kg^−1^) of GSH samples from batch 2 (20.97 mg L^−1^) before and after the cadmium adsorption in fixed-bed columns connected *in series*. The experimental conditions were a pH of 6.2, a flow rate of 140 mL min^−1^, and a temperature of 55 °C. The results are expressed as the dry weight and are the average of three repetitions.

Metal	GSH before Adsorption (mg kg^−1^)	GSH after Adsorption (mg kg^−1^)	Removal (%)
Cadmium (Cd)	139 ± 3.8	14 ± 0.4	90.0
Calcium (Ca)	643 ± 5.3	368 ± 17.7	34.3
Magnesium (Mg)	600 ± 15.9	190 ± 22.5	68.4
Iron (Fe)	40 ± 1.5	9 ± 0.3	81.9
Copper (Cu)	219 ± 8.0	23 ± 0.5	89.7
Zinc (Zn)	77 ± 2.8	15 ± 0.8	78.6
Sodium (Na)	16,000 ± 870.0	12,900 ± 451.5	19.4

**Table 5:** Aminoacidic composition (g (100 g)^−1^) of the GSH sample from batch 2 (20.97 mg L^−1^) before and after the cadmium adsorption in fixed-bed columns connected *in series*. The experimental conditions were a pH of 6.2, a flow rate of 140 mL min^−1^, and a temperature of 55 °C. The results are expressed as the dry weight and are the average of three repetitions.

Aminoacidic Composition	GSH before Adsorption (g (100 g)^−1^)	GSH after Adsorption (g (100 g)^−1^)
Aspartic acid	3.13 ± 0.130 a	3.09 ± 0.121 a
Serine	8.03 ± 0.310 b	5.59 ± 0.123 b
Glutamic acid	12.23 ± 0.220 a	10.32 ± 0.187 a
Glycine	0.23 ± 0.010 a	0.38 ± 0.009 a
Histidine	9.86 ± 0.150 b	12.35 ± 0.121 b
Arginine	8.68 ± 0.230 a	8.31 ± 0.121 a
Threonine	2.48 ± 0.080 b	1.98 ± 0.021 b
Alanine	3.42 ± 0.070 b	2.71 ± 0.052 b
Proline	2.72 ± 0.050 a	2.23 ± 0.041 a
Cysteine	0.12 ± 0.003 a	0.13 ± 0.002 a
Tyrosine	1.77 ± 0.020 a	2.10 ± 0.027 a
Valine	2.71 ± 0.031 a	2.16 ± 0.028 a
Methionine	1.38 ± 0.040 a	1.42 ± 0.029 a
Lysine	4.39 ± 0.081 b	3.06 ± 0.072 b
Isoleucine	2.42 ± 0.056 a	1.87 ± 0.034 a
Leucine	5.37 ± 0.123 b	3.48 ± 0.071 b
Phenylalanine	2.40 ± 0.054 a	1.60 ± 0.036 a

The average values and the standard errors are presented (*n* = 3). Different letters refer to significant differences (*p* < 0.05, Tukey’s test) of the mean values of each amino acid before and after the cadmium removal.

**Table 6:** Data analysis of cadmium removal using 3 columns connected *in series* for scaling up a real process. A cadmium concentration in the GSH of 20.97 mg·L^−1^ and a flow rate of 2800 L h^−1^ (average from the fishing plant) were used.

Parameter	Value	Unit
**Data for calculations**		
Resin packaged per column	140	kg
N° of columns	3	kg
Total resin	420	kg
Flow rate (*)	2800	L h^−1^
Total solids of liquid of GSH (*)	15.0	%
Cd initial concentration (w.w)	21.0	mg L^−1^
*In series* total Cd removal (**)	90	%
Final conc. of Cd (w.w) (**)	2.1	mg L^−1^
Density of liquid GSH (*)	1.1	g mL^−1^
Total solids of dried GSH (*)	95	%
Cation exchange capacity of resin (***)	30	g cation L^−1^
**Parameter calculated**		
Cd final concentration (d.w)	13.9	mg kg^−1^
Cd final conc. in powder (5% of moisture)	13.3	mg kg^−1^
Mass of Cd inlet	58,716.0	mg Cd h^−1^
Mass of Cd removed by the resin	52,844.4	mg Cd h^−1^
Mass of Cd outlet the column	5871.6	mg Cd h^−1^
Level of resin exhaustion by Cd	0.589	%

Notes: (*) are data averages provided by the fishery company; (**) are averages of data from this study; (***) was information from the resin supplier (datasheet).

## References

[1] FAO (2020). The State of World Fisheries and Aquaculture 2020.

[2] Wang W.X., Fisher N.S. (1998). Accumulation of trace elements in a marine copepod. Limnol. Oceanogr..

[3] Raimundo J., Vale C., Rosa R. (2014). Trace element concentrations in the top predator jumbo squid (*Dosidicus gigas*) from the Gulf of California. Ecotoxicol. Environ. Saf..

[4] Sasaki T., Araki R., Michihata T., Kozawa M., Tokuda K., Koyanagi T., Enomoto T. (2015). Removal of cadmium from fish sauce using chelate resin. Food Chem..

[5] Fernandes D., Benianno M.J., Porte C. (2008). Hepatic levels of metal and metallothioneins in two commercial fish species of the Northern Iberian shelf. Sci. Total Environ..

[6] Xiong C.H., Yao C.P. (2009). Study on the adsorption of cadmium(II) from aqueous solution by D152 resin. J. Hazard. Mater..

[7] Yelebe Z.R., Yelebe B.Z., Samuel R.J. (2013). Design of fixed bed column for the removal of metal contaminants from industrial wastewater. J. Eng. Appl. Sci..

[8] Purkayastha D., Mishra U., Biswas S. (2014). A comprehensive review on Cd(II) removal from aqueous solution. J. Water Process. Eng..

[9] Seki H., Okada I., Maruyama H., Kawabe M., Nakade A. (2006). Removal of Cadmium from Squid Liver Method by Competitive Adsorption Method. Aquac. Sci..

[10] Volesky B. (2003). Sorption and Biosorption.

[11] Calderón C., Levio-Raiman M., Diez M.C. (2021). Cadmium removal for marine food application: Comparative study of different adsorbents. Int. J. Environ. Sci. Technol..

[12] Vijayalakshmi K., Sangeetha K., Sudha P.N. (2017). Analysis of packed bed adsorption column with nanochitosan/sodium alginate/microcrystalline cellulose bead for copper (II) removal from aqueous solution. IOSR J. Pharm..

[13] Inglezakis V.J., Loizidou M.D., Grigoropoulou H.P. (2002). Equilibrium and kinetic ion exchange studies of Pb^2+^, Cr^3+^, Fe^3+^ and Cu^2+^ on natural clinoptilolite. Water Res..

[14] Thomas H.C. (1944). Heterogeneous Ion Exchange in a Flowing System. J. Am. Chem. Soc..

[15] Nazari G., Abolghasemi H., Esmaieli M., Sadeghi Pouya E. (2016). Aqueous phase adsorption of cephalexin by walnut shell-based activated carbon: A fixed-bed column study. Appl. Surf. Sci..

[16] Saleh A.D., Sirhan M.M., Ismail A.S. (2020). Study sorption and desorption of Cd^+2^, Pb^+2^ ions by selected chelating resin to removal them from industrial and environmental wastes. Energy Rep..

[17] Taha M.H., Masoud A.M., Khawassek Y.M., Hussein A.E.M., Aly H.F., Guibal E. (2020). Cadmium and iron removal from phosphoric acid using commercial resins for purification purpose. Environ. Sci. Pollut. Res..

[18] Perić J., Trgo M., Vukojević Medvidović N. (2004). Removal of zinc, copper and lead by natural zeolite—A comparison of adsorption isotherms. Water Res..

[19] Elbadawy H.A., Abdel-Salam A.H., Khalil T.E. (2021). The impact of an Amberlite XAD-16-based chelating resin for the removal of aqueous Cd(II) and Pb(II)ions. Microchem. J..

[20] Lázaro N., López Sevilla A., Morales S., Marqués A.M. (2003). Heavy metal biosorption by gellan gum gel beads. Water Res..

[21] Corami A., Mignardi S., Ferrini V. (2008). Cadmium removal from single- and multi-metal (Cd + Pb + Zn + Cu) solutions by sorption on hydroxyapatite. J. Colloid Interface Sci..

[22] Vašák M., Meloni G. (2011). Chemistry and biology of mammalian metallothioneins. J. Biol. Inorg. Chem..

[23] Levio-Raiman M., Briceño G., Schalchli H., Bornhardt C., Diez M.C. (2021). Alternative treatment for metal ions removal from acid mine drainage using an organic biomixture as a low cost adsorbent. Environ. Technol. Innov..

